# Retraction: TRIM29 promotes the progression of colorectal cancer by suppressing EZH2 degradation

**DOI:** 10.3389/ebm.2024.10243

**Published:** 2024-06-10

**Authors:** EBM Editorial Office

Following publication, concerns were raised about the duplicate submission of the article cited above in Experimental Biology and Medicine and BMC Gastroenterology. The article was published on 14 October 2023.

On 20 March 2024 the Editor in Chief of BMC Gastroenterology made the Editor in Chief of EBM aware that the article had been submitted to BMC on 14 March 2023. The article was rejected from BMC.

In line with the evidence of misconduct and the EBM dual submission policy, this article is retracted.

The authors received communication regarding the retraction but did not respond. This communication has been recorded by the publisher.

